# A plea for extension of the anatomical nomenclature: Vessels

**DOI:** 10.17305/bjbms.2020.5256

**Published:** 2021-04

**Authors:** David Kachlik, Vladimir Musil, Alzbeta Blankova, Zuzana Marvanova, Jakub Miletin, Daniela Trachtova, Vlasta Dvorakova, Vaclav Baca

**Affiliations:** 1 Department of Anatomy, Second Faculty of Medicine, Charles University, Prague, Czech Republic; 2 Department of Health Care Studies, College of Polytechnics Jihlava, Jihlava, Czech Republic; 3 Centre of Scientific Information, Third Faculty of Medicine, Charles University, Prague, Czech Republic

**Keywords:** Anatomical terminology, anatomical nomenclature, Terminologia Anatomica, vessels, artery, vein

## Abstract

This article is the fourth and last part of a series aimed at extending and correcting the anatomical nomenclature. Because of the rapid development of internet and the use of electronic formats in communication in anatomy, embryology, histology, medical education, and clinical medicine, an appropriate, precise, and concise anatomical nomenclature is required. Such tool enables to avoid any potential confusion and possible scientific/medical mistakes. The up-to-date official anatomical terminology, Terminologia Anatomica, is available longer than 20 years and needs to be refined and extended. The authors have collected and listed 210 terms and completed them with definitions and/or explanations. We aimed to start a discussion about their potential incorporation into the new revised version of the Terminologia Anatomica. This article is primarily focused on the vessels of the human body (arteries, veins, and lymphatic system).

## INTRODUCTION

This article is the fourth and last part of a series aimed at extending and correcting the anatomical nomenclature. It closes a set of contributions to extent and revise the technical norm for naming morphological structures of the human body in relation to the anatomical nomenclature of the nervous system and senses [1], locomotor system [2], and organs [3]. All the general statements and discussions concerning the history, grammar, and clinical relevance of the anatomical nomenclature and terminology are parts of our previous set of articles. We have also repetitively stressed the importance of anatomical nomenclature in enabling clear, unanimous, and unambiguous communication among specialists. All revised or newly proposed terms to be potentially incorporated into the only official valid version of the anatomical nomenclature called Terminologia Anatomica (TA) are summarized in these articles [1-15].

International Federation of Associations of Anatomists (IFAA) is the only organ responsible for worldwide valid official terminology in human anatomy, histology, and embryology. Concerning the anatomical nomenclature, its last version is quite old-fashioned as it was issued already 22 years ago, in 1998 [16], by not anymore existing Federative Committee on Anatomical Terminology (FCAT), which was in 2005 renamed to Federative International Committee on Anatomical Terminology (FICAT) and in 2009 replaced by Federative International Programme on Anatomical Terminology (FIPAT). Now, FIPAT prepares a new edition called Terminologia Anatomica 2, which is already available online as a draft not yet approved by IFAA and thus not official [17]. The anatomical terminology of vessels is also part of the Terminologia Histologica, published in 2008 [18], Terminologia Embryologica in 2013 [19] and its revision Terminologia Embryologica 2, issued in 2017 [20], and concerning the brain and sensory organs as a part of the Terminologia Neuroanatomica, published in 2017 [21].

The authors have gathered anatomical terms of vascular system absent in the Terminologia Anatomica that they have encountered during their scientific and educational work. Some terms listed here are mentioned and explained in classical textbooks and familiar to all anatomist and thus they are not completed with references. Other terms have been reviewed, refined, or proposed *de novo* for anatomical structures which were previously not well described and/or defined.

Terms presented ***in bold italics*** are newly created terms proposed for incorporation into the Terminologia Anatomica, terms presented in *plain italics* are already listed in the Terminologia Anatomica, terms within quotation marks are non-recommended or obsolete, terms in parentheses are eponyms, synonyms, or explanations, and terms marked with asterisks have been already stated in some of our previous works. In total, 210 terms are suggested for incorporation into the TA: 22 concerning general terms in *Systema cardiovasculare*, 47 items in heart, 56 arteries, 51 veins, and 34 structures in *Systema lymphaticum*. The list of Latin terms compared to their English equivalents is presented in Table 1.

**TABLE 1:**
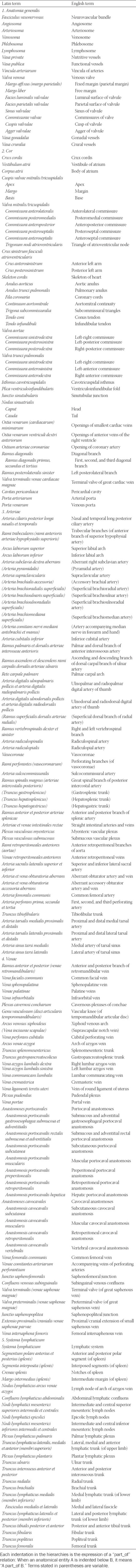
List of Latin terms with their English equivalents

## ANATOMIA GENERALIS

- Fasciculus vasonervosus* (neurovascular bundle) is a bundle of a nerve and one or more vessels. The peripheral or cranial nerve is accompanied by an artery and one or two veins, or, if located in a superficial compartment, a cutaneous nerve is accompanied by a superficial vein. The bundle is encompassed in a fibrous sheath and consists of homonymous or heteronymous structures (*nervus ulnaris – vasa ulnaria*; *nervus fibularis profundus* – *vasa tibialia anteriora*) [11].
- Angiosoma* is an anatomical unit of tissue composed of skin, subcutaneous tissue, fascia, muscle, and bone which is nourished by a specific artery and drained by specific veins [22]. The whole human body consists of 40 angiosomes [23]. ***Arteriosoma*** is such anatomical unit supplied by a specific artery [24] and venosoma* is the same unit drained by a specific vein. In case the extent of the venous drainage is different from arterial supply of the *angiosoma*, the anatomical unit is termed phlebosoma* [25]. The anatomical unit drained by superficial lymphatic vessels is then termed ***lymphosoma*** [13,26].
- Some organs (lungs, liver) feature two types of circulation – nutritive and functional. It is necessary to denominate them also in Latin and there exist terms: The ***vasa privata*** for the nutritive circulation and the ***vasa publica*** for the functional one [27].
- ***Vincula arteriarum*** are thin fibrous bands fixating arteries (with accompanying vessels) to adjacent tissue, e.g. *vasa tibialia anteriora*, hidden in fibrous sheath, to the *membrana interossea cruris*.
- General term for the venous valve *–* *valvula venosa –* is not precise as a classical venous valve comprises two swallow-nest-shaped cusps, called *valvulae*, and that is why the term has to be redressed to the ***valva venosa*** consisting of two *valvulae*. These valvules are attached to the venous wall by a firm ***margo affixus* (*margo parietalis*)** and their free concave ***margo liber*** protrudes into the lumen of the vein. The blood current travels on the ***facies luminalis valvulae***, when it is stopped and turns back, the ***margo liber*** is deflected from the wall toward the opposite valvule, and the lumen is closed. A space formed then by the ***facies parietalis valvulae*** is termed the ***sinus valvulae***. A junction between the *margo affixus* and the *margo liber* is denominated the ***cornu valvulae*** and the slightly elevated parts between adjacent ends of the *margines affixi* are termed the ***commissurae valvae*** as they connect the two valvules. The body of the valvule is called the ***cuspis valvulae*** and it is thickened at the *margo affixus* in the double-horseshoe-shaped ***agger valvulae*** (clinically frequently called “tuberculum” or “limbus”) [28].
- ***Vasa gonadalia*** is a general term which can be used either during the early development when the gender of the embryo is still indifferent or if referred to the *vasa testicularia* in male or to the *vasa ovarica* in female unspecificly, i.e. when general features of these vessels are discussed, not related to the gender.
- ***Vasa cruralia*** is a general term for the principal trunks of the leg (*vasa tibialia anteriora, vasa tibialia posteriora*, and *vasa fibularia*) and can be used when they are considered as general vessels coursing within the leg and supplying the leg and foot.

## COR

- ***Crux cordis*** is an area on the *facies posterior cordis* where the *sulcus coronarius* and the *sulcus interventricularis posterior* meet.
- In pathology, the division of the heart atrium into the ***corpus atrii*** and the ***vestibulum atrii*** is used. Both parts of the atrium are smooth due to the absence of the *musculi pectinati*. On the right side, *musculi pectinati* are overlapping from the auricle onto the free atrial wall and they divide the *atrium dextrum* into the *vestibulum atrii* and the *corpus atrii*. On the left side, the *musculi pectinati* are reduced to the auricle only and thus, the *vestibulum atrii* and the *corpus atrii* are in direct continuation. The *corpus atrii sinistri* receives the *venae pulmonales* (comprises the *ostia venarum pulmonalium*) and the *vestibulum atrii sinistri* is a smooth part of the atrium below this level.
- *Cuspis valvae mitralis/tricuspidalis* features three parts: The ***apex***, the ***margo*,** and the ***basis***.
- Each leaflet (*cuspis*) of a cuspidal valve (*valva mitralis et tricuspidalis*) is separated from the other(s) by commissures: The ***commissura anterolateralis*** and the ***commissura posteromedialis*** are present in the *valva* *mitralis* between its two leaflets and they are denominated according to their location; the ***commissura anteroposterior*** the ***commissura posteroseptalis*** and the ***commissura anteroseptalis*** are present in the *valva tricuspidalis* between its three leaflets and they are denominated according to which leaflets they connect.
- ***Trigonum nodi atrioventricularis*** (of Koch) is a triangle in the right atrium and is defined by three angles: The *ostium sinus coronarii*, the *tendo* *valvulae venae cavae inferioris* (of Todaro) and the *cuspis posterior valvae tricuspidalis* (or more precisely its *commissura anteroseptalis*). Underneath its surface, the *nodus atrioventricularis* is situated.
- *Crus sinistrum fasciculi atrioventricularis* (of Tawara) terminates by a bifurcation into the ***crus anterosinistrum*** and ***crus posterosinistrum***.
- ***Skeleton cordis*** is a general term for the fibrous scaffold supporting the valves and muscle fibers and electrically isolating the atria from the ventricles. It comprises four anuli, two triangles, three ligaments, *pars membranacea septi*, and some other parts [29].
  - ***Anulus aorticus*** et ***anulus trunci pulmonalis*** are integral parts of the *skeleton cordis*, encircling the corresponding ostia, and forming scaffold for corresponding valves.
  - ***Fila coronaria*** (“subvalvar collar”; “subvalvar membrane”) are fibrous subendocardial cords, extensions of the *trigona fibrosa*, forming approximately 75% of the *anuli fibrosi* of the heart skeleton (the rest is only a less distinct sheet of fibroelastic tissue).
  - *Pars membranacea septi* is a small fibrous part of the *skeleton cordis* which contributes to the separation of the right and left atria (***subpars interatrialis partis membranacae septi***) and of the right atrium and left ventricle (***subpars atrioventricularis partis membranaceae septi***). Due to its complex structure, its subparts deserve their own denominations.
  - ***Septum intervalvulare ventriculi dextri*** (“subaortic curtain”) is a part of the fibrous *skeleton cordis* having the shape of a sheet that spans the gap between the fibrous arches supporting the *valvula semilunaris sinistra et dextra valvae aorticae*. It passes caudally within the wall of the right atrium and blends with the fibrous core of the *cuspis anterior valvae mitralis*.
  - ***Continuum aortomitrale*** (“fibrous aortic-mitral continuity”) is a fibrous area between the *trigonum fibrosum dextrum et sinistrum* (laterally), the *anulus fibrosus sinister* (ventrally), and the *anulus aorticus* (dorsally) and is important in aortic valvular replacement surgery.
  - ***Trigona subcommissuralia*** (interleaflets triangles) are located between the *valvulae semilunares* *valvae* *aortae* and their commissures and should be left free in aortal valvuloplasty.
  - ***Tendo coni*** is an inconsistent fibrous band at the contact point of the beginning of the *truncus pulmonalis* and *aorta ascendens*, extending somewhere between the *commissura sinistrodextra valvae aortae* and the pulmonary valvar sinuses.
  - ***Tendo infundibuli*** is an inconstant fibrous band within the *crista supraventricularis*, arising at the level of the *pars membranacea septi interventricularis* and extending cranially to connect with the posterior surface of the *conus arteriosus* (“infundibulum; pars glabra”) at the base of the *truncus pulmonalis*.
  - *Commissurae valvularum semilunarium* are three in each outflow valve and should be distinguished according to their position. *Valva aortae* contains the ***commissura sinistrodextra, commissura posterosinistra*** et ***commissura posterodextra***; *valva trunci pulmonalis* contains the ***commissura sinistrodextra, commissura anterosinistra*** et ***commissura anterodextra***.
- ***Isthmus cavotricuspidalis*** is a fibrous tissue in the lower part of the right atrium, located between the *vena cava inferior* and the *valva tricuspidalis*.
- ***Plica ventriculoinfundibularis*** is a fine muscular bundle interposed between the leaflets of an atrioventricular (cuspidal) and a ventriculoarterial (semilunar) valve, i.e. it separates the inlet (inflow part) of the ventricle from its outlet (outflow part). The *plica* is a relatively fine sheet of a muscle that is folded back on itself [30].
- ***Junctio sinutubularis*** is a narrow zone (2-3.5 mm high) of the *aorta ascendens* above the *bulbus aortae* where the normal tubular configuration of the aorta is attained.
- *Nodus sinuatrialis* can be subdivided into the ***caput*** and the ***cauda***.
- All the vascular openings in the heart cavities are termed *ostia* except the smallest ones, *foramina venarum minimarum*. The term *foramen* is in heart related to the developmental structures (*foramen ovale, foramen primum, foramen secundum*, and *foramen interventriculare*) and that is why the term for openings of the *venae cardiacae minimae* should be changed to the ***ostia venarum (cardiacarum) minimarum***. Similarly, terms for innominate openings of the *venae ventriculi dextri anteriores* should be created – ***ostia venarum ventriculi dextri anteriorum***.
- ***Ostium arteriae coronariae*** is the opening of a coronary artery located in the lateral wall of the *sinus aortae* (of Valsalva).
- ***Ramus diagonalis*** is a clinically used term for the *ramus lateralis*, branch from the *ramus interventricularis anterior*. Its course is oblique (or diagonal) across the ventral surface of the left ventricle toward the *apex cordis* and due to the anatomical position and the preference by clinicians, the term *ramus diagonalis* should be preferred. Sometimes, it can be doubled or tripled (***ramus diagonalis primus, secundus et tertius***).
- ***Ramus posterolateralis sinister*** is a variant largest *ramus posterior ventriculi sinistri* (a terminal branch of the *ramus circumflexus arteriae coronariae sinistrae*), being present in the case of dominance of the *arteria coronaria sinistra* (in that case a bypass can be applied to the stenotic *ramus posterolateralis sinister*).
- ***Valva terminalis venae cardiacae magnae*** (of Vieussens) is a nearly constant (75%) ostial valve at the transition between the *vena cardiaca magna* and the *sinus coronarius*, located at the *margo sinister cordis*.
- ***Cavitas pericardiaca*** is a serous cavity between the *lamina parietalis pericardii* and the *lamina visceralis pericardii*, containing a small amount of the *liquor pericardii*.
- ***Porta arteriarum*** (obsolete term “vagina serosa arteriarum”) is a transition of the *lamina parietalis pericardii* into the *lamina visceralis pericardii*, encompassing both the *aorta ascendens* and the *truncus pulmonalis* in the extent of 2 cm. ***Porta venarum*** (obsolete term “vagina serosa venarum”) is a similar transition on the *venae cavae* and the *venae pulmonales*, located caudally to the former in the posterior wall of the pericardium.

## ARTERIAE

- *Arteriae ciliares posteriores longae* are two in each eyeball and can be distinguished as the medial ***arteria ciliaris posterior longa nasalis*** and the lateral ***arteria ciliaris posterior longa temporalis***.
- ***Rami trabeculares*** are branches from the *ramus anterior* *arteriae hypophysialis superioris*, descending in front of the *infundibulum* and terminating in a large arterial stem, the *arteria trabecularis*, along the *pars tuberalis hypophysis*.
- ***Arcus labiorum superior*** is an arterial anastomosis of the *arteria labialis superior dextra et sinistra* located in the mass of the *musculus orbicularis oris* of the upper lip. Similarly, ***arcus labiorum inferior*** is an arterial anastomosis of the *arteria labialis inferior dextra et sinistra* in the lower lip.
- Arteria subclavia dextra aberrans (ASDA)* is a clinically relevant but rather rare variant (approximately 1% of cases) of the *arteria subclavia*, ramifying as the very last branch from the *arcus aortae*, left (distally) to the origin of the *arteria subclavia sinistra*, and crossing the midline to the right side. It may run in front of the trachea as the *ASDA pretrachealis* (5% of all *ASDA*), between the trachea and the esophagus as the *ASDA retrotrachealis* (15%) and between the esophagus and the vertebral column as the *ASDA retrooesophgea* (retro-esophageal right subclavian artery/RRESA/), found in about 80% of all *ASDA*). In the two latter cases, it could compress the esophagus and may cause problems with swallowing termed dysphagia lusoria that is why in the case of present clinical symptoms, the variant artery used to be called the “arteria lusoria” [11,31].
- ***Arteria pyramidalis*** is a variant branch from the distal part of the *arteria thyroidea superior*, just before its bifurcation into its *ramus anterior et posterior*, supplying the *lobus pyramidalis glandulae thyroideae*, when present (40% of cases) [32].
- ***Arteria supraclavicularis*** is a smaller branch either from the *arteria transversa cervicis* directly [10] or from its *ramus superficialis*, coursing within the *regio cervicalis lateralis* and supplying the fascia and the skin above and below the clavicle.
- The variations of the main upper limb arterial trunks occur in about 20% of cases. They comprise trunks with superficial course, high origins of the forearm trunks, variant vessels or combinations (arteria brachialis superficialis, arteria brachialis accessoria, arteria brachioradialis superficialis, arteria brachioulnaris superficialis, arteria brachioulnoradialis superficialis, arteria brachiomediana superficialis, arteria comitans nervi mediani manus et antebrachii*, etc.) [12,33-42].
- ***Arteria cubitalis inferior*** (obsolete terms “arteria antebrachialis volaris superficialis; arteria antebrachialis mediana”) is the first branch of the *arteria radialis* and is the largest perforating artery in the forearm. It passes superficially between the *musculus brachioradialis* and *musculus pronator teres* and its branches spread across the *fascia antebrachii* along the medial aspect of the *vena cephalica* toward the *processus styloideus radii*. The area of the skin fed by this artery is considered to be the largest nourished by a single cutaneous arterial perforator [43-44].
- *Arteria interossea anterior* bifurcates into two terminal branches: The ***ramus palmaris*** supplying the *musculus pronator quadratus*, running deep underneath, and joining the *rete carpale palmare*; and ***ramus dorsalis*** penetrating the *membrana interossea anterior* to anastomose with the *arteria interossea posterior* which then joins the *rete carpale dorsale*.
- *Ramus carpalis dorsalis arteriae ulnaris* (obsolete term “dorsal ulnar artery”) bifurcates into the ***ramus ascendens et descendens***, the latter anastomosing with the *ramus profundus arteriae ulnaris* [45].
- ***Rete carpale palmare*** is a small anastomotic network on the anterior aspect of the wrist, fed by the *ramus carpalis palmaris arteriae radialis, ramus carpalis palmaris arteriae ulnaris*, and *ramus palmaris arteriae interosseae anterioris*.
- The thumb is fed by four arteries, two dorsal and two palmar, coursing along the nerves in neurovascular bundles. The palmar arteries are larger and nearly constant, the dorsal are smaller and rather variable. Their terminology is not coined and Miletin et al. proposed the descriptive terms ***arteria digitalis ulnopalmaris pollicis*** *et* ***arteria digitalis radiopalmaris pollicis*** for the palmar arteries and ***arteria digitalis ulnodorsalis pollicis*** *et* ***arteria digitalis radiodorsalis pollicis*** for the palmar arteries. Based on the statistics, the current terminology using the term *arteria princeps pollicis* is not specific enough as the word princeps describes the principal (largest) source artery which rather varies for the thumb – the largest caliber features the *arteria metacarpalis palmaris prima* – that is why the term “*arteria princeps pollicis*” should be abandoned and removed from the TA [46].
- An inconstant artery branches from the *arteria radialis* at the dorsum of the hand just before it enters the space between the heads of the *musculus interosseus dorsalis primus*. This artery then runs distally on the dorsal surface of the muscle and at the distal margin of the first web space, it turns back into the palm and forms an anastomosis with the *arcus palmaris superficialis*. Miletin et al. reported its incidence (12%) and proposed the term ***ramus superficialis dorsalis arteriae radialis*** [47].
- *Arteria spinalis anterior* is an unpaired vessel originating as a confluence of a short paired innominate artery branching from the *arteria vertebralis*. These short transverse vessels can be denominated as the ***ramus vertebrospinalis dexter et sinister*.**
- *Arteria medullaris segmentalis* is a term of the TA replacing the clinically used ***arteria radiculospinalis***; another vessel, ***arteria radiculopialis***, is important in clinical medicine and gives off the pial ***vasocoronae***, encompassing the spinal cord horizontally and emanating the ***rami perforantes*** to supply the white matter; finally, the *arteria spinalis anterior* branches off the ***arteriae sulcocommissurales*** into the *fissura mediana anterior* to supply the grey matter [48,49].
- *Arteria radicularis magna* (listed in TNA [21]) or the “artery of Adamkiewicz” is the largest and clinically the most important *ramus spinalis* *arteriae intercostalis posterioris* and thus it should be termed the ***ramus spinalis magnus (arteriae intercostalis posterioris)***.
- The variations of the *truncus coeliacus* are quite frequent and its incomplete formation appears in 8% of cases as ***truncus gastrosplenicus*** (3.46%), ***truncus hepatosplenicus*** (3.88%), and ***truncus hepatogastricus*** (0.24%) [50].
- *Arteria splenica* bifurcates into the ***ramus anterior et posterior*** ***arteriae splenicae***, which then further ramify into the segmental *rami splenici*.
- The mighty vasculature of the intestine consists of source arteries (and draining veins), their main macroscopic branches (and tributaries), fine vessels supplying the intestinal wall called ***arteriae*** et venae intestinales rectae* and then by intramural plexuses. Their terminology should match the nervous counterparts, i.e. ***plexus vasculosus myentericus*** located between the *stratum longitudinale* and *stratum circulare tunicae muscularis* and ***plexus vasculosus submucosus*** located with the submucosa [3,51].
- Rami retroperitoneales anteriores* are direct fine branches from the *aorta abdominalis*, the *arteria renalis* and the *arteria renalis accessoria*, the *arteria testicularis* or the *arteria ovarica*, and the *arteria iliaca communis*. They feed the adjacent lymph nodes (*nodi lymphatici lumbales et iliaci communes*), ureter, peritoneum, loose connective tissue around the *aorta abdominalis* and the *vena cava inferior*, autonomic nervous plexuses and their ganglia and the vascular wall of both the *aorta abdominalis* and *vena cava inferior* forming their *vasa vasorum*. These *rami* are followed by homonymous fine veins (***venae retroperitoneales anteriores***), emptying into the ventral aspect of the *vena cava inferior* and its tributaries [52,53].
- *Arteriae sacrales laterales* are usually two branches (***arteria sacralis lateralis superior*** et ***inferior***) emanating from the *divisio posterior arteriae iliacae internae*, descending and bifurcating into the corresponding *rami spinales* to enter four *foramina sacralia anteriora ossis sacri* to supply the bone, contents of the *canalis sacralis* and adjacent part of the deep back muscles.
- The *arteria obturatoria* is usually a branch from the *arteria iliaca interna*, but sometimes it can ramify from the *arteria epigastrica inferior*. In such case, it should be termed ***arteria obturatoria aberrans***. If there are two arteries present, one originating from the *arteria iliaca interna* and the other from the *arteria epigastrica inferior*, they should be called as the ***arteria obturatoria propria*** and ***arteria obturatoria acesssoria aberrans***, respectively. Similar approach can be applied to the veins: ***Vena obturatoria aberrans*** and ***vena obturatoria accessoria aberrans***.
- In angiology and vascular surgery, the proximal segment of the *vasa femoralia*, from the arbitrary beginning under the *ligamentum inguinale* within the *lacuna vasorum* to the branching of the *arteria profunda femoris* (and termination of the *vena profunda femoris*, respectively), are termed the arteria femoralis communis* and the vena femoralis communis*. The distal segment is then in vascular surgery termed “arteria et vena femoralis superficialis,” but phlebologists do not accept this terminology as they consider superficial veins only those coursing above the layer of muscular fascia, e.g. *venae saphenae* [4-6,13,54], which can be agreed to.
- Three branches of the *arteria profunda femoris*, supplying the posterior and medial groups of the thigh muscles are called the *arteriae perforantes* and classified using numbers according to the level of their origin as the arteria perforans prima*, arteria perforans secunda*, and arteria perforans tertia*. Their accompanying veins are denominated in a bit different way as the venae comitantes arteriarum perforantium* due to the fact that the term *venae perforantes* is reserved for the veins interconnecting the superficial and deep venous systems [9,13,15,54].
- Truncus tibiofibularis* is the short proximal segment of the *arteria tibialis posterior*, between its origin from the *arteria poplitea* to the branching point of the *arteria fibularis* [13].
- *Arteriae tarsales mediales* should be specified as the arteria tarsalis medialis proximalis* and the arteria tarsalis medialis distalis*; *arteriae tarsales laterales* as the arteria tarsalis lateralis proximalis* and the arteria tarsalis lateralis* [13].
- ***Arteria sinus tarsi medialis*** (“arteria canalis tarsi; arteria of Salvi”) and ***arteria sinus tarsi lateralis*** (“arteria anastomotica tarsi; ramus anastomicus tarsi; perforating vessel of sinus tarsi”) are important feeding arteries of the talus with variable origin from the *arteria tibialis posterior* (or less often from the *arteria plantaris medialis*), and from the *arteria dorsalis pedis* (or less often from the *arteria tarsalis lateralis proximalis* or *arteria malleolaris lateralis anterior*), respectively [13,55].

## VENAE

- *Vena retromandibularis* terminates in a specific way by a bifurcation (similarly to the *vena portae hepatis* and the *vena dorsalis penis/clitoridis profunda*) into the ***ramus anterior*** draining into the *vena facialis* and the ***ramus posterior*** forming the *vena jugularis externa* by the confluence with the *vena auricularis posterior*.
- ***Vena facialis communis*** is a short terminal segment of the *vena facialis* after it is joined by the *ramus anterior venae retromandibularis*; then, it usually drains into the *vena jugularis interna*.
- Some tributaries of the *plexus pterygoideus* are missing in the TA: The ***vena sphenopalatina***, the ***venae palatinae***, the ***vena infraorbitalis***, and the ***plexus cavernosi concharum***, especially in the area of the *concha nasalis inferior*.
- *Zona bilaminaris* (of Rees) [56], also called “retroarticular/retrodiscal plastic pad/cushion (of Zenker)” [57] or “trilaminar zone (of Smeele)” [58], is the posterior continuation of the *discus articularis articulationis temporomandibularis*, consisting of the *stratum superius* (fibroelastic loose network of elastic and collagen fibers, adipose tissue and fine vessels, attached to the posterior margin of the *fossa mandibularis* *ossis temporalis*), *stratum inferius* (stiff/non-elastic network of collagen fibers, attached to the *caput mandibulae*), and in between interposed ***genu vasculosum*** (adipose tissue, connective tissue and mainly a venous plexus, a dorsal extension of the *plexus pterygoideus*), which serves as shock-absorber during the joint movements.
- ***Arcus venosus xiphoideus*** is a transverse venous arch (present in approximately 80%) connecting the *venae thoracicae internae dextrae et sinistrae* across the midline, located ventral to the *symphysis xiphosternalis* [59].
- ***Vena incisurae scapulae*** is a variable vein (58%), originating on the *facies costalis scapulae*, below the *incisura scapulae*, either from a vein accompanying the nutrient artery of the scapula and/or veins located beneath the fascia of the *musculus subscapularis*. It passes through the *incisura scapulae* and drains into the *vena suprascapularis* immediately after passing the notch [60,61].
- Vena perforans cubitalis* (of Gracz) is the thickest perforating vein of the upper limb, located in the *fossa cubitalis*, usually connecting the *vena mediana cubiti* and the *venae radiales* and present in 100% of cases [12,62].
- ***Truncus splenomesentericus*** is the last segment of the *vena splenica*, between its confluence with the *vena mesenterica inferior* (present in approximately 60-70% of cases) and the beginning of the *vena portae hepatis* [63].
- ***Truncus gastropancreatocolicus*** (of Henle) is a short venous trunk, formed usually by the confluence of the *vena gastroomentalis dextra*, the *vena pancreaticodudenalis superior anterior*, and the *vena colica dextra superior*, draining into the *vena mesenterica superior* and located behind the *caput pancreatis*. It is present in 87% of cases [64].
- *Vena azygos* originates usually by the confluens of the *vena lumbalis ascendens dextra* and the *vena subcostalis dextra* and immediately receives the variant ***vena azygos lumbalis dextra***, branching from the posterior aspect of the *vena cava inferior* at the level of the opening of the *vena lumbalis secunda* (often a common trunk with the *vena lumbalis secunda dextra* or the *vena renalis dextra*) and present in 34% of cases. *Vena hemiazygos* receives a corresponding contralateral variant vessel – ***vena azygos lumbalis sinistra*** – branching from the posterior aspect of the *vena renalis sinistra* and present in 28% of cases [65]. *Vena renalis sinistra* features rather frequently (64% of cases) a communicating vein dorsally into the retroperitoneal tissue, either the *vena azygos lumbalis sinistra* draining into the *vena hemiazygos* or the ***vena communicans lumbalis*** emptying into the upper *venae lumbales* or into the *vena lumbalis ascendens sinistra* (64% of cases) [66,67].
- ***Vena cremasterica*** is a gentle vein accompanying the *arteria cremasterica* in male and draining into the *vena epigastrica inferior*. ***Vena ligamenti teretis uteri*** is a gentle vein accompanying the homonymous artery in female [8].
- Plexus pudendus* (of Santorini) is a small pelvic venous plexus located within the lower part of the *spatium retropubicum* (of Retzius) behind the inferior part of the *symphysis pubica* and in front of the inferior part of the urinary bladder and on the anterior and inferolateral surfaces of the prostate [8,13,54].
- *Vena portae hepatis* is the only vessel with the non-concordant adjective “portae” and thus the specifying word “hepatis” can be omitted and the term for the main vessel of the liver can be shortened to ***vena portae***.
- Under pathological condition called the portal hypertension, veno-venous bypasses (shunts) open to relieve hypertension in the system of the *vena portae*. These are enlarged naturally formed veno-venous anastomoses in specific locations and they deserve to be officially termed as the ***anastomoses portocavales***. They comprise the following major routes:
  - ***Anastomosis portocavalis gastrooesophagea*** ***(submucosa et adventitialis)*** *–* between tributaries of the *vena gastrica sinistra* and the *venae oesophageae* including both the submucous and adventitial levels, sometimes presenting as esophageal and paraesophageal varices;
  - ***Anastomosis portocavalis rectalis*** ***(submucosa et adventitialis)*** *–* between tributaries of the *vena rectalis superior* and the *venae rectales mediae et inferiores* including both the submucous and adventitial levels, the former sometimes presenting as internal hemorrhoids;
  - ***Anastomosis portocavalis subcutanea*** *–* between the *venae paraumbilicales* (of Sappey) and tributaries of the *venae epigastricae superficiales et venae thoracoepigastricae* of both sides, presenting rather more rarely as “caput Medusae;”
  - ***Anastomosis portocavalis muscularis*** *–* between the *venae paraumbilicales* (of Sappey) and tributaries of the *venae epigastricae inferiores et superiores* of both sides within the *musculus rectus abdominis*;
  - ***Anastomosis portocavalis preperitonealis*** (veins of Burrow) *–* between the *venae paraumbilicales* (of Sappey) and tributaries of the *plexus venosus vesicalis* running in the midline along the *ligamentum umbilicale medianum*;
  - ***Anastomosis portocavalis retroperitonealis*** (veins of Retzius) *–* between veins of the spleen and veins within the *mesenterium* and/or the *mesocola*, and the retroperitoneal veins and veins of the posterior abdominal wall (tributaries to the *venae suprarenales, renales, tesiculares/ovaricae, lumbales, phrenicae inferiores et iliolumbales*);
  - ***Anastomosis portocavalis hepatica*** *–* between veins of the hepatic capsule and veins of the diaphragm in the extent of the *area nuda hepatis*.

- Similarly to the portocaval anastomoses, under pathological condition with obturated *vena cava superior* or *vena cava inferior*, veno-venous bypasses (shunts) open to relieve the hypertension in the system of one of the *venae cavae*, termed the ***anastomoses cavocavales***, which can be classified into the following groups:
  - ***Anastomosis cavocavalis subcutanea*** *–* between tributaries of the *venae epigastricae superficiales* and those of the *venae thoracoepigastricae* within the subcutaneous layer of the anterolateral trunk wall;
  - ***Anastomosis cavocavalis muscularis*** *–* between tributaries of the *venae epigastricae inferiores* and those of the *venae epigastricae superiores* within the *musculus rectus abdominis*;
  - ***Anastomosis cavocavalis retroperitonealis*** *–* between tributaries of the *venae lumbales* and those of the *venae lumbales ascendentes* draining into the *vena azygos* and *vena hemiazygos*;
  - ***Anastomosis cavocavalis vertebralis*** *–* between tributaries of the *plexus venosi vertebrales (externi et interni*) (plexus of Batson) extending along the *columna vertebralis* and within the *canalis vertebralis*.

- Many new terms have been added concerning the lower limb veins, mainly concerning the superficial and perforating veins interconnecting the superficial and deep venous systems (*venae perforantes*). Among others, it is necessary to emphasize the following:
  - The termination of the *vena saphena magna* into the *vena femoralis communis* is called the junctio saphenofemoralis* and it is an integral part of the confluens venosus subinguinalis* (“crosse; bulbus; venous star of Paturet”), bordered by two valves of the *vena saphena magna*: The valva terminalis* (situated 1-2 mm distal to the *junctio saphenofemoralis*) and the valva preterminalis* (located 3-5 cm distally). This confluence receives the centripetal segments of the smaller venous tributaries: *Vena epigastrica superficialis*, *vena circumflexa ilium superficialis, vena pudenda externa superficialis, vena saphena magna accessoria anterior et posterior*, and *vena circumflexa femoris anterior*. Similarly, the term junctio saphenopoplitea* is applied to the termination of the *vena saphena parva* into the *vena poplitea* [9,13,54].
  - Extensio proximalis/cranialis venae saphenae parvae* (“vena femoropoplitea of Hyrtl;” “extensio cranialis venae saphenae parvae”) is a proximal continuation of the *vena saphena parva*. It ascends from the *fossa* *poplitea* on the posterior aspect of the thigh and terminates in more variants: It can submerge as the *vena perforans femoris posterior*/*posterolateralis* and drain into the *vena profunda femoris*; it can terminate in the muscular or subcutaneous venous plexus; it can continue as the vena intersaphena femoris* (of Giacomini) and drain into the *vena saphena magna* or its tributaries; or rarely, it can ascend as high as the gluteal region and empty into the *venae gluteae inferiores*. The *extensio proximalis/cranialis* is present in approximately 95% of cases [6,13,68,69].
  - *Venae perforantes* of the lower limb are numerous communications between the superficial and deep systems. Their extensive nomenclature has been proposed in 2005 and explained in detail by our team in 2019. The major impact consists in the rule that eponyms should be replaced with systemic terminology, e.g. first Cockett’s perforator with the vena perforans cruris tibialis posterior inferior* [15,70].

## SYSTEMA LYMPHATICUM

Generally, the term “lymphoid” meaning from the linguistic point of view precisely “similar to lymph” (“eidos” is a Greek term for the form) should be abandoned and instead the term “lymphatic” should be preferred in all terms related to the lymph, i.e. also in the denomination of the whole chapter: ***Systema lymphaticum***.

- Spleen can be divided into segments separated by avascular planes; constant are polar segments: ***Segmentum polare anterius et polare posterius***, and variable is/are central segment(s): ***Segmentum interpositum / segmenta interposita***.
- ***Crenae splenis*** are deep clefts or notches (reaching some 2-3 cm in depth) located predominantly on the *margo superior splenis* (former “margo crenatus”) and *facies diaphragmatica* *splenis* [71].
- ***Margo intermedius (splenis)*** is a ridge separating the spleen surface for the kidney and that for the stomach.
- ***Nodus lymphaticus arcus venae azygoi*** is a lymph node from the group of the *nodi lymphatici bronchopulmonales* situated in the concavity of the ***arcus venae azygoi***, the terminal segment of the *vena azygos* turning above the right lung hilum. It has to be stressed that the genitive of the Greek word azygos is azygoi.
- ***Confluens lymphaticus abdominalis*** describes the very variable confluens of lymphatic *trunci lumbales et intestinales*, sometimes forming the *cisterna chyli*, located retroperitoneally approximately at the level of the first or second lumbar vertebra.
- Lymph nodes draining the small and large intestine are arranged in groups which can be classified into three or four rows/levels, respectively. The small intestine lymph nodes comprise: *Nodi lymphatici juxtaintestinales* located close to the intestinal wall, ***nodi lymphatici mesenterici superiores intermedii*** located along the jejunal and ileal vessels, and ***nodi lymphatici mesenterici superiores centrales*** situated around the *radix mesenterii* along the trunk of the *arteria mesenterica superior*. The large intestine lymph nodes consist of four rows/levels: ***Nodi lymphatici epicolici*** stuck closely to the intestinal wall, *nodi lymphatici paracolici* located along the *arteria marginalis coli* (of Drummond), ***nodi lymphatici mesenterici inferiores intermedii*** located along the colic vessels (and classified in detail according to certain vessels: *Nodi ileocolici, appendiculares, colici dextri, colici medii, colici sinistri, sigmoidei, et* *rectales superiores*), and finally ***nodi lymphatici mesenterici inferiores centrales*** situated along the trunk of the *arteria mesenterica inferior*.
- The lymphatic trunks of the limb (also known as “collectors”) can be divided into the superficial and deep, the former running independently on the superficial veins, and the latter extending in intimate relation to the deep vascular bundles. The term “*lymphaticus*” should be preferred to its grammatically incorrect synonym “*lymphoideus*” – see above [4,10].
- Three main superficial lymphatic trunks of the upper limb constitute from the plexus lymphaticus palmaris* on the palmar aspect of the digits and hand: Truncus lymphaticus lateralis membri superioris* travels on the lateral side of the forearm and arm and empties into the *nodi lymphatici axillares* or directly into the *plexus lymphaticus axillaris* and *nodi lymphatici cervicales lateralis profundi*; truncus lymphaticus medialis membri superioris* courses on the medial side of the forearm and arm and empties into the *nodi lymphatici axillares*; and truncus lymphaticus anterior membri superioris* runs on the ventral side of the forearm and empties into one of the former trunks [12].
- Three main superficial lymphatic trunks of the upper limb constitute from the plexus lymphaticus plantaris* on the inferior aspect of the toes and sole: Truncus lymphaticus medialis membri inferioris* ascends in front of the *malleolus medialis* on the ventromedial aspect of the leg dividing into the fasciculus medialis* (traveling medially to the *condylus medialis femoris*) and the fasciculus lateralis* and drains into the *nodi lymphatici inguinales superficiales*; truncus lymphaticus lateralis membri inferioris* courses on the lateral aspect of the leg and usually drains into the *nodi lymphatici inguinales superficiales*; and truncus lymphaticus posterior membri inferioris* runs on the posterior aspect of the leg and drains into the *nodi lymphatici poplitei profundi* [13].
- The deep lymphatic trunks are termed according to the blood vessel they accompany: Truncus ulnaris*, truncus radialis*, ***truncus interosseus anterior*** et posterior*, and truncus brachialis*; ***truncus tibialis posterior*** et anterior*, truncus fibularis*, truncus popliteus*, and truncus femoralis*. In the pelvis, the situation is more complicated, see [13].

## DISCUSSION

Some anatomically and/clinically very important terms have been already mentioned in our previous articles, but we felt inevitable to remind readers of them [6,8,9,11-13,15]. If we check the anatomical terminology in current journals, monographs, and textbook, there are still many terms found to be obsolete, incorrect, or even eponymic although the last revision of the anatomical nomenclature – Terminologia Anatomica (TA) – has been issued more than 20 years ago and eponyms have been banned from the anatomical nomenclature already in the Parisiensia Nomina Anatomica (PNA) in 1955 [16,72-76].

If we check the new proposal of Terminologia Anatomica 2 (TA 2), posted online [17] as a not yet approved version (the approval is planned at the next IFAA meeting in Istanbul in 2022), there are not many changes concerning the vessels, but there are some substantial changes concerning the heart which should be thoroughly reviewed and considered if they are appropriate and if they have a chance to be accepted by clinicians [e.g., change of the *sulcus interventricularis posterior* (including the *ramus interventricularis posterior*) to “sulcus interventricularis inferior” (and “ramus interventricularis inferior”)].

The main task of every anatomist and all anatomical societies, which continues and never stops, is to cultivate, clean, and revise the anatomical nomenclature not only in the anatomical field, education, journals, and textbooks but above all among clinicians, physicians, secondary school teachers, as well as lay public not only in English and Latin but also in individual languages.

## References

[1] Kachlik D, Musil V, Baca V (2017). A plea for extension of the anatomical nomenclature. Part 1:Nervous system and senses. Folia Morphol (Warsz).

[2] Musil V, Blankova A, Baca V (2018). A plea for an extension of the anatomical nomenclature:The locomotor system. Bosn J Basic Med Sci.

[3] Musil V, Blankova A, Dvorakova V, Turyna R, Baca V (2019). A plea for an extension of the anatomical nomenclature:Organ systems. Bosn J Basic Med Sci.

[4] Kachlik D, Baca V, Bozdechova I, Cech P, Musil V (2008). Anatomical terminology and nomenclature:Past, present and highlights. Surg Radiol Anat.

[5] Kachlik D, Bozdechova I, Cech P, Musil V, Baca V (2009). Mistakes in the usage of anatomical terminology in clinical practice. Biomed Pap.

[6] Kachlik D, Pechacek V, Baca V, Musil V (2010). The superficial venous system of the lower extremity:New nomenclature. Phlebology.

[7] Kachlik D, Pechacek V, Musil V, Baca V (2010). Information on the changes in the revised anatomical nomenclature of the lower limb veins. Biomed Pap.

[8] Kachlik D, Pechacek V, Musil V, Baca V (2010). The venous system of the pelvis:New nomenclature. Phlebology.

[9] Kachlik D, Pechacek V, Musil V, Baca V (2012). The deep venous system of the lower extremity:New nomenclature. Phlebology.

[10] Kachlik D, Musil V, Baca V (2015). Terminologia Anatomica after 17 years:Inconsistencies, mistakes and new proposals. Ann Anat.

[11] Kachlik D, Musil V, Baca V (2016). Contribution to the anatomical nomenclature concerning general anatomy and anatomical variations. Surg Radiol Anat.

[12] Kachlik D, Musil V, Baca V (2017). Contribution to the anatomical nomenclature concerning upper limb anatomy. Surg Radiol Anat.

[13] Kachlik D, Musil V, Baca V (2018). Contribution to the anatomical nomenclature concerning lower limb anatomy. Surg Radiol Anat.

[14] Musil V, Sach J, Kachlik D, Patzelt M, Stingl J (2018). Vasa vasorum:An old term with new problems. Surg Radiol Anat.

[15] Kachlik D, Pechacek V, Hnatkova G, Hnatek L, Musil V, Baca V (2019). The venous perforators of the lower limb - A new terminology. Phlebology.

[16] Florida Comprehensive Assessment Test (1998). Terminologia Anatomica.

[17] Florida Comprehensive Assessment Test (2019). Terminologia Anatomica.

[18] Florida Comprehensive Assessment Test (2008). Terminologia Histologica:International Terms for Human Cytology and Histology.

[19] Florida Comprehensive Assessment Test (2013). Terminologia Embryologica:International Embryological Terminology.

[20] Florida Comprehensive Assessment Test (2017). Terminologia Embryologica.

[21] Florida Comprehensive Assessment Test (2017). Terminologia Neuroatomica. Federative International Programme for Anatomical Terminology.

[22] Taylor GI, Palmer JH (1987). The vascular territories (angiosomes) of the body experimental-study and clinical-applications. Br J Plast Surg.

[23] Brodmann M (2013). The Angiosome Concept in Clinical Practise. http://www.evtoday.com/2013/05/the-angiosome-concept-in-clinical-practice.

[24] Cariou JL (1995). 1984-1994: Dix ans de lambeaux cutanés les progrès et évolutions conceptuels. Ann Chir Plast Esthet.

[25] Caggiati A (2016). The Vascular architecture. Phlebosomes do they exist?. J Theor Appl Vasc Rres.

[26] Suami H (2017). Lymphosome concept: Anatomical Study of the lymphatic system. J Surg Oncol.

[27] Von Hayek H (1953). Die menschliche Lunge.

[28] Varga I, Kachlik D, Klein M (2020). A plea for extension of the official nomenclature of the microscopic structure of human tissues and organs, the terminologia histologica. Folia Morphol (Warsz).

[29] Saremi F, Sanchez-Quintana D, Mori S, Muresian H, Spicer DE, Hassani C (2017). Fibrous skeleton of the heart: Anatomic overview and evaluation of pathologic conditions with CT and MR imaging. Radiographics.

[30] Hosseinpour AR, Jones TJ, Barron DJ, Brawn WJ, Anderson RH (2007). An appreciation of the structural variability in the components of the ventricular outlets in congenitally malformed hearts. Eur J Cardio-Thorac.

[31] Polguj M, Chrzanowski L, Kasprzak JD, Stefanczyk L, Topol M, Majos A (2014). The aberrant right subclavian artery (arteria lusoria):The morphological and clinical aspects of one of the most important variations-a systematic study of 141 reports. Sci World J.

[32] Mangalgiri A, Mahore D, Kapre M (2018). Pyramidal artery:An artery to pyramidal lobe-a new nomenclature. Indian J Otolaryngol Head Neck Surg.

[33] Kachlik D, Hajek P, Konarik M, Krchov M, Baca V (2016). Coincidence of superficial brachiomedian artery and bitendinous palmaris longus:A case report. Surg Radiol Anat.

[34] Kachlik D, Konarik M, Baca V (2011). Vascular patterns of upper limb:An anatomical study with accent on superficial brachial artery. Bosn J Basic Med Sci.

[35] Kachlik D, Konarik M, Hajek P (2011). A case of a double variant of the arterial system in the upper extremity:Arteria brachialis accessoria et arteria comitans nervi mediani. Arch Biol Sci.

[36] Kachlik D, Konarik M, Riedlova J, Baca V (2016). Brachiomedian artery (arteria brachiomediana) revisited:A comprehensive review. Bosn J Basic Med Sci.

[37] Konarik M, Kachlik D, Baca V (2014). A coincidental variation of the axillary artery:The brachioradial artery and the aberrant posterior humeral circumflex artery passing under the tendon of the latissimus dorsi muscle. Bosn J Basic Med Sci.

[38] Konarik M, Knize J, Baca V, Kachlik D (2009). Superficial brachioradial artery (radial artery originating from the axillary artery):A case-report and its embryological background. Folia Morphol (Warsz).

[39] Konarik M, Musil V, Baca V, Kachlik D (2020). Upper limb principal arteries variations:A cadaveric study with terminological implication. Bosn J Basic Med Sci.

[40] Rodriguez-Niedenfuhr M, Sanudo JR, Vazquez T, Nearn L, Logan B, Parkin I (1999). Median artery revisited. J Anat.

[41] Rodriguez-Niedenfuhr M, Vazquez T, Nearn L, Ferreira B, Parkin I, Sanudo JR (2001). Variations of the arterial pattern in the upper limb revisited:A morphological and statistical study, with a review of the literature. J Anat.

[42] Rodriguez-Niedenfuhr M, Vazquez T, Parkin I, Sanudo RJ (2003). Arterial patterns of the human upper limb:Update of anatomical variations and embryological development. Eur J Anat.

[43] Lamberty BG, Cormack fGC (1982). The forearm angiotomes. Br J Plast Surg.

[44] Magden O, İçke Ç, Arman C, Atabey A (1997). An anatomical study of the inferior cubital artery. Eur J Plast Surg.

[45] Malkoc I, Karagoz H, Alp BF, Gundogdu C, Diyarbakir S (2012). Anatomical evaluation of the dorsal ulnar artery. Turk J Med Sci.

[46] Miletin J, Sukop A, Baca V, Kachlik D (2017). Arterial supply of the thumb:Systemic review. Clin Anat.

[47] Miletin J, Sukop A, Baca V, Kachlik D (2020). Innominate variant artery in the first web space. Ann Anat.

[48] Miyasaka K, Asano T, Ushikoshi S, Hida K, Koyanagi I (2000). Vascular anatomy of the spinal cord and classification of spinal arteriovenous malformations. Interv Neuroradiol.

[49] Bosmia AN, Hogan E, Loukas M, Tubbs RS, Cohen-Gadol AA (2015). Blood supply to the human spinal cord:Part I. Anatomy and hemodynamics. Clin Anat.

[50] Whitley A, Oliverius M, Kocian P, Havluj L, Gurlich R, Kachlik D (2020). Variations of the celiac trunk investigated by multidetector computed tomography:Systematic review and meta-analysis with clinical correlations. Clin Anat.

[51] Kachlik D, Baca V, Stingl J (2010). The spatial arrangement of the human large intestinal wall blood circulation. J Anat.

[52] Turyna R, Kachlik D, Kucera E, Kujal P, Feyereisl J, Baca V (2013). Complications in right-sided paraaortic lymphadenectomy:Ventral tributaries of the inferior vena cava. J Anat.

[53] Turyna R, Kachlik D, Feyreisl J, Stingl J, Baca V (2014). Anterior retroperitoneal rami:Until now unnamed direct branches of the abdominal aorta. Clin Anat.

[54] Caggiati A, Bergan JJ, Gloviczki P, Jantet G, Wendell-Smith CP, Partsch H (2002). Nomenclature of the veins of the lower limbs:An international interdisciplinary consensus statement. J Vasc Surg.

[55] Mulfinger GL, Trueta J (1970). The blood supply of the talus. J Bone Joint Surg Br.

[56] Rees LA (1954). The structure and function of the mandibular joint. Br Dent J.

[57] Zenker W (1956). Das retroarticuläre plastische Polster des Kiefergelenkes und seine mechanische Bedeutung. Z Anat Entwicklungsgesch.

[58] Smeele LE (1988). Ontogeny of relationship of middle ear and temporomandibular (squamomandibular) joint in mammals [corrected]. I. Morphology and ontogeny in man. Acta Anat (Basel).

[59] Loukas M, Tobola MS, Tubbs RS, Louis RG, Karapidis M, Khan I (2007). The clinical anatomy of the internal thoracic veins. Folia Morphol (Warsz).

[60] Podgórski M, Sibiński M, Majos A, Stefańczyk L, Topol M, Polguj M (2014). The suprascapular vein:A possible etiology for suprascapular nerve entrapment and risk of complication during procedures around the suprascapular foramen region. Orthop Traumatol Surg Res.

[61] Al-Redouan A, Holding K, Kachlik D (2020). “Suprascapular canal”:Anatomical and topographical description and its clinical implication in entrapment syndrome. Ann Anat.

[62] Gracz KC, Ing TS, Soung LS, Armbruster KF, Seim SK, Merkel FK (1977). Proximal forearm fistula for maintenance hemodialysis. Kidney Int.

[63] Benninger B (2013). Splenomesenteric vein:Formally recognising a clinically relevant section of the portal venous drainage system. Folia Morphol.

[64] Stefura T, Kacprzyk A, Droś J, Pędziwiatr M, Major P, Hołda MK (2018). The venous trunk of henle (gastrocolic trunk):A systematic review and meta-analysis of its prevalence, dimensions, and tributary variations. Clin Anat.

[65] Bergman RA, Tubbs RS, Shoja MS, Loukas M (2016). Bergman's Comprehensive Encyclopedia of Human Anatomic Variation.

[66] Yao Y, Okada Y, Yamato M, Ohtomo K (2003). Communicating vein between the left renal vein and left ascending lumber vein:Incidence and significance on abdominal CT. Radiat Med.

[67] Yi SQ, Ueno Y, Naito M, Ozaki N, Itoh M (2012). The three most common variations of the left renal vein:A review and meta-analysis. Surg Radiol Anat.

[68] Cavezzi A, Labropoulos N, Partsch H, Ricci S, Caggiati A, Myers K (2006). Duplex ultrasound investigation of the veins in chronic venous disease of the lower limbs--UIP consensus document. Part II. Anatomy. Eur J Vasc Endovasc Surg.

[69] Sagoo KS, Vari R, Helmdach M, Salfeld K (2004). Chirurgie der Vena giacomini. Phlebologie.

[70] Caggiati A, Bergan JJ, Gloviczki P, Eklof B, Allegra C, Partsch H (2005). Nomenclature of the veins of the lower limb:Extensions, refinements, and clinical application. J Vasc Surg.

[71] Yildiz AE, Ariyurek MO, Karcaaltincaba M (2013). Splenic anomalies of shape, size, and location:Pictorial essay. Sci World J.

[72] Woerdeman MW (1957). Nomina anatomica parisienssia (1955) et BNA (1895).

[73] Strzelec B, Chmielewski PP, Gworys B (2017). The Terminologia Anatomica matters:examples from didactic, scientific, and clinical practice. Folia Morphol (Warsz).

[74] Chmielewski PP, Strzelec B (2020). Should Terminologia Anatomica be revised and extended?A critical literature review. Folia Morphol (Warsz).

[75] Chmielewski PP (2020). New Terminologia Anatomica highlights the importance of clinical anatomy. Folia Morphol (Warsz).

[76] Chmielewski PP, Domagala ZA (2020). Terminologia anatomica and its practical usage:Pitfalls and how to avoid them. Folia Morphol (Warsz).

